# Revision of *Tipula* (*Yamatotipula*) *stackelbergi* Alexander (Diptera, Tipulidae), and a short discussion on subspecies among crane flies

**DOI:** 10.3897/zookeys.162.2216

**Published:** 2012-01-05

**Authors:** Jukka Salmela

**Affiliations:** 1 Zoological Museum, Department of Biology, FI-20014 University of Turku, Finland

**Keywords:** Palaearctic region, crane flies, *Tipula*, new synonyms

## Abstract

All available type material of *Tipula stackelbergi* Alexander, *Tipula usuriensis* Alexander and *Tipula subpruinosa* Mannheims were examined. *Tipula (Yamatotipula) stackelbergi* **stat. rev.** is elevated from a subspecies of *Tipula (Yamatotipula) pruinosa* Wiedemann to a valid species. Two new synonyms are proposed: *Tipula usuriensis* **syn. n.** proved to be a junior synonym of. *Tipula (Yamatotipula) pruinosa* and *Tipula subpruinosa* **syn. n.** a junior synonym of *Tipula (Yamatotipula) freyana* Lackschewitz. *Tipula (Yamatotipula) stackelbergi* is redescribed, male and female terminalia of *Tipula (Yamatotipula) pruinosa* are illustrated and discussed. Female terminalia of *Tipula (Yamatotipula) freyana* are described and illustrated for the first time. A key to both sexes of *Tipula (Yamatotipula) stackelbergi* and *Tipula (Yamatotipula) pruinosa*, and a key to females of *Tipula (Yamatotipula) chonsaniana*, *Tipula (Yamatotipula) freyana* and *Tipula (Yamatotipula) moesta* are provided. Subspecies are not uncommon among crane flies, but their ranges and traits are poorly known. An interdisciplinary approach (genetics, ecology, taxonomy) is suggested if subspecific ranks are to be used in tipuloid systematics.

## Introduction

The description of *Tipula stackelbergi* (Diptera, Tipulidae) was based on male holotype collected from Russian East Siberia (Alexander 1934). Later this species was also recorded from the Russian Far East (Savchenko 1961; Pilipenko 2009). Savchenko (1961) considered *Tipula stackelbergi* as a subspecies of *Tipula pruinosa* Wiedemann, based on the small structural differences between the two taxa. He also transferred *Tipula stackelbergi* to the subgenus *Tipula* (*Yamatotipula)* Matsumura. In the same publication, Savchenko suggested two synonyms for *Tipula stackelbergi*, namely *Tipula usuriensis* Alexander, 1925 and *Tipula subpruinosa* Mannheims, 1954. However, both synonyms were uncertain because Savchenko did not examine the type material of these two species. The description of *Tipula usuriensis* was based on male holotype, collected from Siberia (exact locality uncertain) and the description of *Tipula subpruinosa* was based on two females, collected from northern Finland (holotype) and Sweden (paratype) (Alexander 1925; Mannheims 1954). Since the taxonomic treatment by Savchenko (1961), both species have remained synonyms of *Tipula stackelbergi* (e.g. Oosterbroek and Theowald 1992) and apparently the type material has remained unstudied. In addition, *Tipula stackelbergi* has been used as an example of a tipulid (sub)species with a large but disjunct range in the Palaearctic region (Oosterbroek et al. 2001).

Subspecies are traditionally held as geographically separate and genetically distinct populations within the species’ range, permitting gene flow in the area of contact (Wilson and Brown 1953; O’Brien and Mayr 1991; Patten and Unitt 2002). Despite possible interbreeding between subspecies, subspecies may retain differences in respective life cycles or other traits (Hewitt 2002; Kothera et al. 2009). Among birds, high subspecies richness was associated with large breeding ranges, island dwelling, inhabiting montane regions, habitat heterogeneity and low latitude; on the other hand, species phylogenetic age was a poor predictor of subspecies richness (Phillimore et al. 2007). Definition of subspecies, and propensity of naming subspecific taxa, vastly differs among taxonomic groups. High proportions of higher plants, mammals and birds have subspecies, less so compared to invertebrates (Haig et al. 2006). New molecular methods have revolutionized subspecific classifications: i) formerly held subspecies gain no support at all, ii) subspecies are proposed to be valid species or iii) their status as operational evolutionary units is supported (Ball and Avise 1992; Patten and Unitt 2002; Tsao and Yeh 2008; Miller et al. 2011). Despite problems in correct recognition and delineation of subspecies, subspecific taxa are seen as powerful tools in conservation and as meaningful biological entities (Haig et al. 2006; Phillimore and Owens 2006).

In crane flies (Diptera, Tipuloidea) subspecific ranks are not uncommon. For example, out of 493 and 168 Palaearctic Tipulidae taxa described by C.P. Alexander (1889–1981) and E.N. Savchenko (1909–1994), respectively, 24 and 26 taxa are currently ranked as subspecies (data from Oosterbroek 2011). However, the recent tendency has been to elevate former subspecies to valid species (Starý 2006; Salmela and Autio 2009; Starý and Brodo 2009). In these cases, former subspecies are clearly separated upon differences in male and female hypopygial structures. In addition, due to the improved faunistic knowledge, range-sizes of former subspecies are in reality much larger than was previously known. On the other hand, some western Palaearctic (sub)species are most probably recent origin of Pleistocene glacial and interglacial periods; examples of such species are present in especially in the Iberian peninsula and Asia minor (Oosterbroek 1980). In general, tipuloid subspecies are elusive and very poorly known, and no rigorous assessment on the suitability of subspecific rank among crane flies has been carried out. Based on subjective opinion, perhaps a majority of the current Palaearctic tipuloid subspecies are in fact valid species. Furthermore, most allopatric or parapatric crane fly populations, that are genetically distinct from nominotypical (sub)species, are still to be found by biologists. Based on above mentioned references, subspecies should not be proposed on exiguous basis, relying on a small number of studied specimens and subtle differences in coloration or other structures. Instead, an interdisciplinary approach (genetics, ecology, taxonomy) is suggested if subspecific ranks are to be on a solid ground.

In this article I present the results of an examination of all available type material of *Tipula stackelbergi*, *Tipula usuriensis* and *Tipula subpruinosa*. I propose changes to the nomenclature of these species and I also review the morphology of *Tipula pruinosa* and *Tipula stackelbergi*, with an emphasis on male and female genitalia. In addition, female genitalia of *Tipula (Yamatotipula) freyana* are illustrated and a key to *Tipula (Yamatotipula) freyana* and females of *Tipula (Yamatotipula) chonsaniana* and *Tipula (Yamatotipula) moesta* are provided.

## Material and methods

The morphological terminology used here mainly follows Alexander and Byers (1981). Terminology of some special parts of male genitalia was taken from Frommer (1963) or is explained in the figures. The following acronyms for museums and collections are used in the text: MZHF – Finnish Museum of Natural History (Zoological Museum), University of Helsinki, Helsinki, Finland; PVM – Private Collection of V.-M. Mukkala, Kaarina, Finland; USNM – Smithsonian Institution, National Museum of Natural History, Washington DC, USA; ZMUC – Zoological Museum, University of Copenhagen, Copenhagen, Denmark; ZMUT – Zoological Museum, University of Turku, Turku, Finland; ZISP – Zoological Institute Russian Academy of Sciences, St. Petersburg, Russia. Due to the courtesy of Valentin Pilipenko (Moscow State University, Russia), I was able study high quality digital photos of male hypopygium of *Tipula (Yamatotipula) pruinosa* (Russia: Moscow, 1 male, Altay, 1 male) and *Tipula (Yamatotipula) stackelbergi* (Russia: Primorski kray, 1 male).

Layer photos were taken using an Olympus SZX16 stereomicroscope attached to an Olympus E520 digital camera. Digital photos were captured using the programmes Deep Focus 3.1 and Quick PHOTO CAMERA 2.3. Layer photos were finally combined with the program Combine ZP.

### Tipula (Yamatotipula) stackelbergi

Alexander stat. rev.

http://species-id.net/wiki/Tipula_stackelbergi

**Figure 1.**
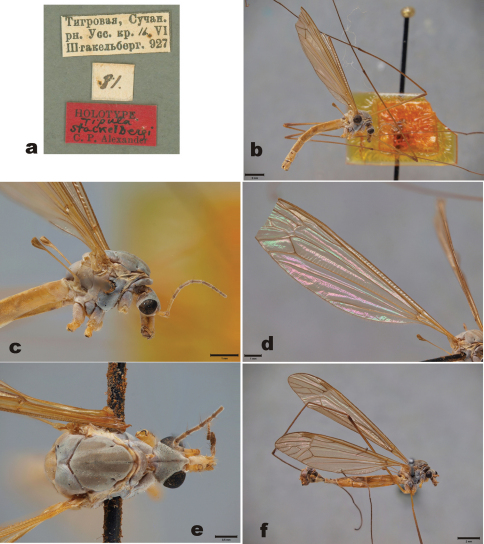
*Tipula (Yamatotipula) stackelbergi* Alexander **a** Label of the holotype **b** Holotype male, habitus, lateral view. Yellowish celluloid board is attached below the specimen; most probably C.P. Alexander dissected hypopygium on this board. The surface of the board is heavily cracked, no details of the hypopygium are discernible **c** Thorax and head, holotype, lateral view **d** Right wing, holotype **e** Thorax and head, holotype, dorsal view **f** Male (Russia, Vladivostok), habitus, lateral view. Scale bars: **b**, **f** 2 mm; **c** & **d** 1 mm; e 0.5 mm.

Figs 1 2 3e 6a, c, d

Tipula (Tipula) stackelbergi Alexander 1934: 305.
Tipula (Yamatotipula) pruinosa *stackelbergi*Savchenko 1961: 292.
Tipula (Yamatotipula) pruinosa stackelbergi Oosterbroek and Theowald 1992: 165.
Tipula (Yamatotipula) pruinosa stackelbergi Oosterbroek 2011: http://nlbif.eti.uva.nl/ccw/

#### Material examined.

Holotype of *Tipula stackelbergi*: male, pinned specimen (ZISP). “Tigrovaja, Suchan./ rn.Uss.kr. 16.VI/ Stackelberg. 927” (white label, partly hand written, in Cyrillic letters). “81” (white label, handwritten). “HOLOTYPE/ Tipula stackelbergi/ C.P. Alexander” (red label, partly handwritten) (Fig. 1a).

With except of the male hypopygium, the holotype specimen is in rather good condition (Figs 1b, c). All legs are detached from the specimen, but four legs are glued to the pin below the specimen. Tips of wings are broken. Right antenna is broken, only scape and pedicel are left; left antenna has seven flagellomeres. Tip of abdomen is broken; apparently hypopygium is mounted on a celluloid strip, which is attached on a pin. The surface of this strip is heavily cracked, and the structure of the hypopygium cannot be examined.

#### Other material.

Russia, Vladivostok, Nekrutenko leg, 2.VI. 1957, 1 male, 1 female (ZISP).

#### Redescription.

Male. Head yellowish brown, with grey pruinosity. Rostrum yellowish, nasus distinct, bearing numerous light hairs. Palpi brown. Scape yellowish, elongate, length 387–450 μm, width 126–131 μm (n=2). Pedicel yellowish, globular, length 147 μm, width 139 μm (n=1). Flagellomere 1 yellowish brown, length 486 μm, width 91 μm (n=1). Flagellomere 2 length 464 μm, width 79 μm (n=1). Flagellomeres bear erect short hairs, giving silvery appearance. Flagellomeres 2–7 elongate, brown, with dark verticils (Fig. 1c).

Prescutum with four brown stripes (Fig. 1e). Pronotum, prescutum, scutum, anepisternum, katepisternum and meron brownish, with grey pruinosity. Scutellum, anepimeron and laterotergite yellowish. Anterior part of mediotergite yellowish, more brownish in posterior part, having two weak longitudinal brown stripes. Coxa 1 brown. Anterior part of coxa 2 brown, posterior part yellow. Coxa 3 yellow. Femorae yellowish brown, darkening toward tarsi. Wings without markings, pterostigma brown (Fig. 1f). Wing length 13.8 mm (n=1). Halter yellowish.

**Figure 2.**
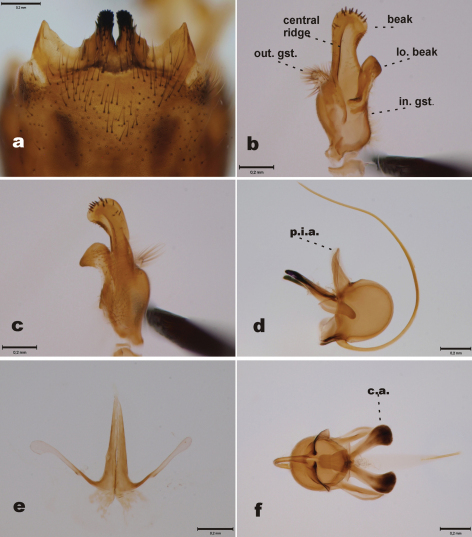
*Tipula (Yamatotipula) stackelbergi* Alexander, male (Russia, Vladivostok) **a** 9^th^ tergite, dorsal view **b** Outer and inner gonostylus, posterior view; abbreviations: out. gst.= outer gonostylus, in. gst. = inner gonostylus, lo. beak = lower beak **c** Inner gonostulys, anterior view **d** Sperm pump, lateral view; abbreviation: p.i.a. = posterior immovable apodeme **e** Aedeagal guide, dorsal view **f** Sperm pump, ventral view; abbreviation: c.a. = compressor apodeme. Scale bars: 0.2 mm.

**Figure 3.**
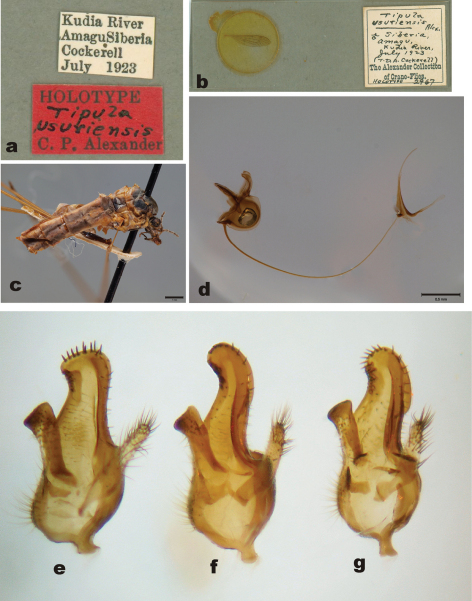
*Tipula usuriensis* Alexander (=syn. of *Tipula (Yamatotipula) pruinosa* Wiedemann), holotype male **a** Label of the holotype **b** Slide mounted wing **c** Habitus, lateral view **d** Sperm pump (lateral view) and aedeagal guide (dorso-lateral view). Scale bars: c 1mm; d 0.5 mm.

Abdominal tergites yellowish brown, slightly darkening toward tip of abdomen. 9^th^ tergite with two median projections, densely covered by dark bristles. Lateral corners of 9^th^ tergite glabrous, pointed (Fig. 2a). 9^th^ sternite with median incision, bearing two fleshy and hairy outgrowths in the margin of the incision. Outer gonostylus worm-like, apical half covered by light hairs (Fig. 2b). Inner gonostylus elongate (Figs 2b, c, 3e); beak rounded, with ten stout apical bristles and four subapical weaker bristles; central ridge with few weak bristles along its length; lower beak roundish, not angular. Posterior immovable apodeme of sperm pump almost straight (Fig. 2d). Distal end of compressor apodeme of sperm pump club-shaped, roundish (Fig. 2f). Aedeagal guide as in Fig. 2e.

Female. In general similar to male. Scutellum brown, abdominal tergites brown. Wing length 18.4 mm (n=1). Female terminalia as in Fig. 6a. Basal part of hypogynial valves with dense black setae. Proximal ends of valves roundish, tapering toward bases (Fig. 6c). Genital fork of vaginal apodeme brown, rather narrow in its whole length (Fig. 6d). Dorsal view of vaginal apodeme as in Fig. 6d.

### Tipula (Yamatotipula) pruinosa

Wiedemann

http://species-id.net/wiki/Tipula_pruinosa

Figs 3a–d, 3f–g 4 6b, e, f

Tipula pruinosa Wiedemann 1817: 64.
Tipula usuriensis Alexander 1925: 18, **syn. n.**
Tipula (Tipula) pruinosa Mannheims 1952: 91.
Tipula (Yamatotipula) pruinosa pruinosa Savchenko 1961: 288.
Tipula (Yamatotipula) pruinosa Oosterbroek and Theowald 1992: 165.
Tipula (Yamatotipula) pruinosa pruinosa Oosterbroek 2011: http://nlbif.eti.uva.nl/ccw/ (for unlisted European references, see Mannheims 1952 and Savchenko 1961).

#### Material examined.

Holotype of *Tipula usuriensis*: male, pinned specimen (USNM). “Kudia River/Amagu Siberia/Cockerell/July 1923” (white label, printed). “HOLOTYPE /Tipula/ usuriensis/ C.P. Alexander” (red label, partly handwritten). Slide, permanently mounted wing. “Tipula usuriensis Alex./ ♀ Siberia, Amagu,/ Kudia River/ July 1923, (T.D.A. Cockerell) / The Alexander Collection of Crane-Flies/HOLOTYPE 2967” (white label, partly handwritten). (Figs 3a, b). The holotype specimen of *Tipula usuriensis* is in quite bad condition (Fig. 3c). Half of the abdomen (distal part) and four legs are glued to a card. One wing (length 14.0 mm) is slide mounted and one wing is glued to a white card, one leg is also glued to the same card. Scape, pedicel and three flagellomeres of antennae are present. The holotype is also laterally flattened, perhaps due to compression of the freshly collected specimen. Hypopygium was detached by the author from the cardboard, macerated in KOH and finally preserved in glycerol in a microvial.

#### Other material.

Finland. Savonia borealis: Kiuruvesi, Jynkänjärvi 63.5194°N; 26.6941°E, 13.VII. 2008, J. Salmela leg, 2 males (ZMUT); Ostrobottnia australis: Ilmajoki, Kivistönmäki 62.8492°N; 22.6623°E, 1 female, V.-M. Mukkala leg (PVM); Regio aboensis: Taivassalo, Orikvuori 60.6027°N; 21.6653°E, 26.VI. 2005 V.-M. Mukkala leg, 1 female (PVM); Regio aboensis: Turku, Piipanoja 60.4918°N; 22.3017°E, 22.VI. 2011 A. Teräs leg, 1 female, 4 males (ZMUT).

#### Redescription of male and female terminalia.

**Figure 4.**
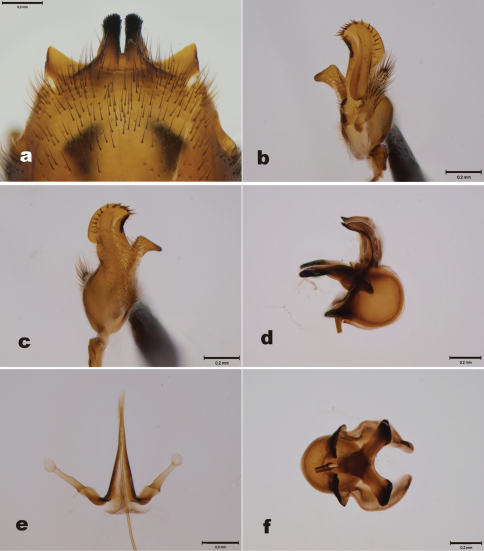
*Tipula (Yamatotipula) pruinosa* Wiedemann, male (Finland, Turku) **a** 9^th^ tergite, dorsal view **b** Outer and inner gonostylus, posterior view **c** Inner gonostulys, anterior view **d** Sperm pump, lateral view **e** Aedeagal guide, dorsal view **f** Sperm pump, ventral view. Scale bars 0.2 mm.

Male. 9^th^ tergite (Fig. 4a) essentially similar to *Tipula (Yamatotipula) stackelbergi*. 9^th^ tergite with two median projections, densely covered by dark bristles, lateral corners of the tergite glabrous, pointed (Fig. 4a). 9^th^ sternite with median incision, bearing two fleshy and hairy outgrowths. Outer gonostylus worm-like, apical half covered by dark hairs (Figs 4b, c). Inner gonostylus elongate. Beak rounded, rather wide, resembling helmet (Figs 4b, c, 3f–g). Apical portion of beak bearing around 20 stout bristles, central ridge with numerous weak bristles, along the whole length of the ridge. Lower beak angular. Posterior immovable apodeme of sperm pump curved in lateral and ventral view (Figs 4d, f). Distal end of compressor apodeme of sperm pump truncated (Fig. 4f). Aedeagal guide as in Fig. 4e.

Female. Female terminalia as in Fig. 6b. Basal part of hypogynial valves with dense black setae, proximal ends of valves rounded, widest sub-basally, not tapering toward proximal end (Fig. 6e). Stalk of genital fork gradually widening toward caudal and proximal ends, being narrowest around midpoint (Fig. 6f). Dorsal view of vaginal apodeme as in Fig. 6f.

Geographical variation: The above mentioned description of male terminalia suites well to European specimens. The beak of the inner gonostylus among specimens from Asia is somewhat more i) sinuous, ii) slender and iii) with fewer stout bristles. Variation related to the geographical origin of the specimens is not detected in the structure of sperm pump. It is likely that *Tipula (Yamatotipula) pruinosa sinapruinosa* Yang & Yang, 1993 is similar to the holotype of *Tipula usuriensis* and to a male from Russia, Altay. These eastern Palaearctic specimens could perhaps be given a subspecific or infrasubspecific rank under *Tipula (Yamatotipula) pruinosa*. However, one widespread species with slight geographic variation in the coloration of head and abdomen (see Alexander 1925; Yang and Yang 1993) and appearance of inner gonostylus is recognized here.

### Tipula (Yamatotipula) freyana

Lackschewitz

http://species-id.net/wiki/Tipula_freyana

Figs 5 7a–c

Tipula freyana Lackschewitz 1936: 292.
Tipula (Tipula) subpruinosa Mannheims 1954: 42, **syn. n.**
Tipula (Yamatotipula) freyana freyana Savchenko 1961: 251.
Tipula (Yamatotipula) freyana Salmela and Autio 2009: 54.

#### Material examined.

**Figure 5.**
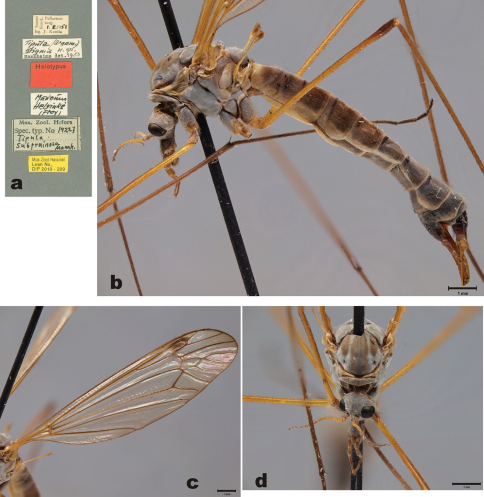
*Tipula subpruinosa* Mannheims (=syn. of *Tipula (Yamatotipula) freyana* Lackschewitz), holotype female **a** Label **b** Habitus, lateral view **c** Left wing **d** Thorax and head, dorsal view. Scale bars: 1 mm.

Holotype of *Tipula subpruinosa*: female, pinned specimen (MZHF). “Suomi/ KemL./ Pallastunturit/ 1.8.1951/ leg J. Kaisila” (white label, partly handwritten; backside: “Pyhäkuru” handwritten). “Tipula (Oreom.)/ stigma n. sp./ Mannheims det. 1953” (white label, partly handwritten). “Holotypus” (red label, printed). “Museum/ Helsinki/ Frey” (white label, handwritten). “Mus. Zool. H:fors/ Spec. typ. No 14227/ Tipula/ subpruinosa Mann.” (grayish label, partly handwritten) (Fig. 5a). Pyhäkuru is located in NW Finland, Muonio, Pallas-Yllästunturit National Park, rough coordinates of the type locality are 68.079°N; 24.083°E.

The holotype specimen is in good condition (Figs 5b, c, d). Left mid leg is missing, other legs are intact. Right wing has minor rupture proximal to the pterostigma, Costa is slightly damaged. Abdominal terminalia of the specimen were detached by me, macerated in KOH and later preserved in glycerol in a microvial. This microvial is attached to the same pin as the specimen. The name “*stigma*” has never been published, and it has most probably been a working title by Mannheims while compiling his first account of Finnish tipulids (Mannheims 1954).

Paratype: female, pinned specimen (ZMUC). “Lpl Sorsele/ Vallnäs tr / 18.7.1925 / S. Gaunitz” (white-gray label, unclear hand writing) “ex coll./ Peder Nielsen” (white label, printed) “Tipula (Tipula) / subpruinosa n sp.) / Mannheims det 1953” (white label, partly handwritten) “Tipula (Tipula) / subpruinosa n sp.) / Mannheims det 1953” (white label, partly handwritten) “Paratypoid” (red label, printed). The paratype specimen is in rather bad condition. Left antenna has nine and right antenna ten segments. All legs are broken, remnants of two legs are glued to a card below the specimen.

Other material. Finland. Karelia borealis: Lieksa, Nurmespuro 63.4030°N; 28.1972°E, 19.VI.–14.VII. 2008, J. Salmela leg, 2 females (ZMUT); Lapponia kemensis pars occidentalis: Kittilä, Palovaara E 68.0054°N; 24.7736°E, 23.VI. 2009 J. Salmela leg, 1 female (ZMUT); Lapponia enontekiensis: Enontekiö, Tarvantovaara, Pahtavaara SE 68.6518°N; 22.5909°E, 11.VI.–19.VII. 2009, J. Salmela leg, 2 males, 1 female.

**Figure 6.**
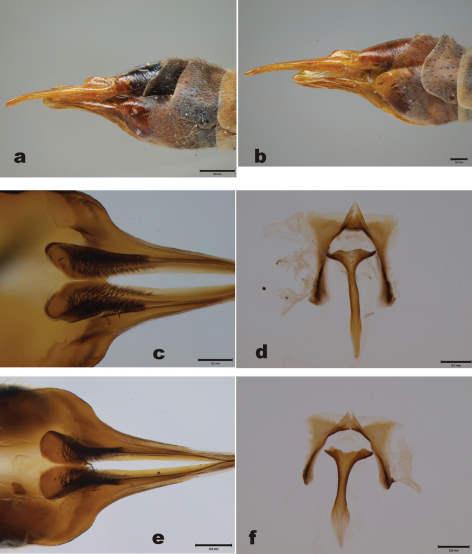
Female terminalia. *Tipula (Yamatotipula) stackelbergi* Alexander (Russia, Vladivostok) **a** Female cerci, lateral view, pinned specimen **c** Hypogynial valves, dorsal view **d** Vaginal apodeme and genital fork, dorsal view. *Tipula (Yamatotipula) pruinosa* Wiedemann (Finland, Turku) **b** female cerci, lateral view, pinned specimen **e** Hypogynial valves, dorsal view **f** Vaginal apodeme and genital fork, dorsal view. Scale bars: a 0.5 mm; b, c, d, e, f 0.2 mm.

**Figure 7.**
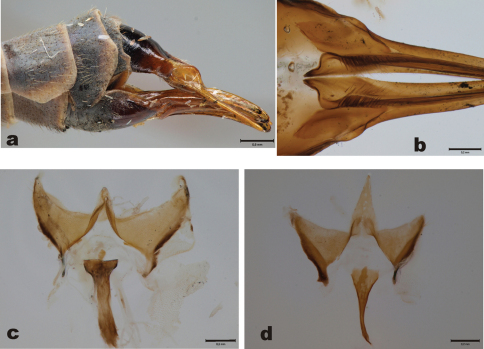
Female terminalia. *Tipula (Yamatotipula) freyana* Lackschewitz (holotype of *Tipula subpruinosa* Mannheims (Finland, Pallastunturit) **a** Female cerci, lateral view, pinned specimen **b** Hypogynial valves, dorsal view **c** Vaginal apodeme and genital fork, dorsal view. *Tipula (Yamatotipula) chonsaniana* Alexander (Finland, Taivalkoski) **d** Vaginal apodeme and genital fork, dorsal view.

#### Description of female terminalia.

Female terminalia as in Fig. 7a. Basal part of hypogynial valves with modest setosity, proximal ends of valves pointed (Fig. 7b). Genital fork of vaginal apodeme dark brown, slightly sinuous in lateral view. Dorsal view of vaginal apodeme and genital fork as in Fig. 7c.

##### Key to the *Tipula (Yamatotipula) pruinosa* and *Tipula (Yamatotipula) stackelbergi*

**Table:** 

1	Males	2
–	Females	3
2	Beak of inner gonostylus relatively wide, helmet-like, with numerous (ca. 20) stout bristles (Figs 4b, c). Sperm pump dark, posterior immovable apodeme curved in lateral and ventral view (Figs 4d, f)	*Tipula (Yamatotipula) pruinosa*
–	Beak of inner gonostylus rather narrow, with ca. 10 stout bristles (Figs 2 b, c). Sperm pump lighter, posterior immovable apodeme almost straight in lateral and ventral view (Figs 2d, f)	*Tipula (Yamatotipula) stackelbergi*
3	Basal part of hypogynial valves widest sub-basally, not tapering toward base (Fig. 6f). Stalk of genital fork gradually widening toward caudal and proximal ends, being narrowest around midpoint (Fig. 6g)	*Tipula (Yamatotipula) pruinosa*
–	Basal part of hypogynial valves roundish, tapering toward base (Fig. 6d). Genital fork rather narrow in its whole length (Fig. 6e)	*Tipula (Yamatotipula) stackelbergi*

##### Key to the females of *Tipula (Yamatotipula) moesta* and related species

**Table:** 

1	Body coloration dark; scape, pedicel and 1^st^ flagellomere dark brown	*Tipula (Yamatotipula) moesta*
–	Body coloration lighter; scape, pedicel and 1^st^ flagellomere yellowish	2
2	Stalk (proximal 2/3) of genital fork very narrow, needle-like (Fig. 7d)	*Tipula (Yamatotipula) chonsaniana*
–	Stalk (proximal 2/3) of genital fork wider, as in Fig. 7c	*Tipula (Yamatotipula) freyana*

## Discussion

In the present paper I suggest three changes to the nomenclature of Palaearctic Tipulidae: i) *Tipula (Yamatotipula) stackelbergi* is a valid species, not a subspecies of *Tipula (Yamatotipula) pruinosa* ii) *Tipula usuriensis* is neither a valid species nor a synonym of *Tipula (Yamatotipula) stackelbergi*, it is instead a junior synonym of *Tipula (Yamatotipula) pruinosa* and iii) *Tipula subpruinosa* is not a synonym of *Tipula (Yamatotipula) stackelbergi*, it is a junior synonym of *Tipula (Yamatotipula) freyana*. It remains questionable whether *Tipula (Yamatotipula) pruinosa sinapruinosa* is a valid subspecies. Based on the original description (Yang and Yang 1993) it is likely that Chinese specimens are conspecific with other eastern Palaearctic *Tipula (Yamatotipula) pruinosa* specimens. If these eastern Palaearctic specimens are to be ranked as subspecies below *Tipula (Yamatotipula) pruinosa*, *Tipula usuriensis* is the oldest available name for the taxon. However, as discussed above, subspecies should be delineated through several criteria, e.g. ecology and genetics. More data on Asian *Tipula (Yamatotipula) pruinosa* populations should be available for the assessment of speciation and reliable use of subspecific rank.

*Tipula (Yamatotipula) pruinosa* and *Tipula (Yamatotipula) stackelbergi* are closely related but valid species. The species pair is well separated due to the differences in male genitalia (see the key to the species), but less so regarding female genitalia. More females of *Tipula (Yamatotipula) stackelbergi* should be studied in order to firmly validate the diagnostic differences presented here. *Tipula (Yamatotipula) stackelbergi* is a very rarely collected species, known only from East Siberia and the Russian Far East (Alexander 1934; Savchenko 1961; Pilipenko 2009).

*Tipula subpruinosa*, described from Finland and Sweden, was thought to be a synonym of *Tipula (Yamatotipula) stackelbergi* (Savchenko 1961; Oosterbroek and Theowald 1992). Due to this tentative synonymy, *Tipula (Yamatotipula) stackelbergi* was erroneously thought to be present in Fennoscandia. However, examination of the holotype of *Tipula subpruinosa* revealed that the species is a junior synonym of *Tipula (Yamatotipula) freyana*, not *Tipula (Yamatotipula) stackelbergi*. Hence, *Tipula (Yamatotipula) stackelbergi* should be removed from the list of European crane flies. It should be noted that the description of *Tipula subpruinosa* was very short and lacking any figures; it is not surprising it led to fallacious interpretation. In a similar vein, *Tipula usuriensis* was also tentatively synononymized by Savchenko (1961) with *Tipula (Yamatotipula) stackelbergi*. In his description of *Tipula usuriensis* Alexander (1925) provided figures depicting male 9^th^ tergite and lateral view of hypopygium, but these figures can now be considered too general to discriminate between *Tipula (Yamatotipula) pruinosa* and *Tipula (Yamatotipula) stackelbergi*.

Compared to *Tipula (Yamatotipula) stackelbergi* and *Tipula (Yamatotipula) pruinosa*, *Tipula (Yamatotipula) freyana* is phylogenetically rather distant to these two species, being instead close to *Tipula (Yamatotipula) moesta* Riedel and *Tipula (Yamatotipula) chonsaniana* Alexander (e.g. Salmela and Autio 2009). Although illustrations of male hypopygium, or parts of it, of *Tipula (Yamatotipula) freyana* have been provided by several authors (see Salmela and Autio 2009), no figures of female terminalia have been hitherto published. A key to the females of *Tipula (Yamatotipula) chonsaniana*, *Tipula (Yamatotipula) freyana* and *Tipula (Yamatotipula) moesta* explains the diagnostic differences between these three species (see above). Figures of female genital forks of *Tipula (Yamatotipula) moesta* and *Tipula (Yamatotipula) chonsaniana* were provided by Salmela and Autio (2009).

## Supplementary Material

XML Treatment for Tipula (Yamatotipula) stackelbergi

XML Treatment for Tipula (Yamatotipula) pruinosa

XML Treatment for Tipula (Yamatotipula) freyana
